# Two cases of combined immunodeficiency with ITPR3 mutations presenting with life-threatening severe EBV-associated hemophagocytic lymphohistiocytosis

**DOI:** 10.3389/fimmu.2025.1653662

**Published:** 2025-09-12

**Authors:** Lang Yu, Yulin Li, Liwen Zhang, Wenhui Li, Ming Sun, Yunfei An, Hao Xiong, Peina Jin, Xiaodong Zhao

**Affiliations:** ^1^ National Clinical Research Center for Child Health and Disorders, Ministry of Education Key Laboratory of Child Development and Disorders, Children’s Hospital of Chongqing Medical University, Chongqing, China; ^2^ Chongqing Key Laboratory of Child Rare Diseases in Infection and Immunity, Children’s Hospital of Chongqing Medical University, Chongqing, China; ^3^ Molecular Medicine Diagnostic and Testing Center, Chongqing Medical University, Chongqing, China; ^4^ Department of Hematology, Wuhan Children’s Hospital, Tongji Medical College, Huazhong University of Science and Technology, Wuhan, Hubei, China; ^5^ Department of Rheumatology and Immunology, Children’s Hospital of Chongqing Medical University, Chongqing, China; ^6^ PICU, The First Affiliated Hospital of Zhengzhou University, Zhengzhou, China

**Keywords:** hemophagocytic lymphohistiocytosis, HLH, ITPR3, EBV, combined immunodeficiency

## Abstract

**Introduction:**

ITPR3 encodes a subunit of the inositol 1,4,5-trisphosphate receptor (IP3R), which forms a Ca^2+^ channel on the endoplasmic reticulum (ER) membrane responsible for ER Ca^2+^ release. Recently, both autosomal dominant and recessive ITPR3 mutations have been reported in association with combined immunodeficiency (CID), accompanied by multisystem manifestations including neurological involvement.

**Methods:**

We retrospectively analyzed the clinical characteristics of two patients with ITPR3 deficiency accompanied by hemophagocytic lymphohistiocytosis (HLH) at our center. Through a literature review, we further compared their clinical manifestations with those of combined immunodeficiency (CID) patients who presented with HLH.

**Results and Discussions:**

Our two CID patients with multisystem disorders harboring germline heterozygous ITPR3 mutations (c.7570C>G, p.Arg2524Gly and c.7570C>T, p.Arg2524Cys). Both patients developed severe Epstein-Barr virus (EBV)-associated HLH, and one patient succumbed to disease-related complications. Our study demonstrates that ITPR3-associated CID confers a susceptibility to EBV-driven pathologies, particularly HLH, which warrants heightened clinical vigilance. Therefore, early hematopoietic stem cell transplantation (HSCT) should be considered to improve survival outcomes in these patients.

## Introduction

Maintenance of cytosolic Ca²^+^ homeostasis is essential for fundamental cellular processes including motility, proliferation, and programmed cell death (1, 2). The electrochemical gradient drives Ca²^+^ influx through store-operated calcium entry (SOCE) mechanisms following depletion of endoplasmic reticulum (ER) Ca²^+^ stores (3, 4). SOCE-mediated Ca²^+^ signaling is particularly crucial for immune regulation and lymphocyte activation. Inherited defects in Ca²^+^ signaling pathways, collectively termed CRAC channelopathies, result from autosomal recessive loss-of-function mutations in STIM1, ORAI1, or CRACR2A that impair SOCE function (5–8). Affected patients present with combined immunodeficiency (CID) accompanied by multisystem manifestations including muscular hypotonia, osteopetrosis, and hypohidrotic ectodermal dysplasia with characteristic dental enamel defects.

In humans, IP_3_ receptors (IP_3_Rs) form tetrameric Ca²^+^ release channels composed of ITPR1, ITPR2, and ITPR3 subunits (9). While dominant ITPR3 mutations were initially associated with Charcot-Marie-Tooth neuropathy, Neumann et al. first established a causal relationship between autosomal recessive ITPR3 mutations and CID, demonstrating the critical role of ER Ca²^+^ flux in immune function (10, 11). Subsequent reports have identified monoallelic dominant-negative ITPR3 mutations in patients with CID and multisystem involvement, expanding the spectrum of Ca²^+^ signaling-related immunodeficiencies (12, 13).

We describe two cases of heterozygous ITPR3 mutations associated with Epstein-Barr virus-induced hemophagocytic lymphohistiocytosis (EBV-HLH), including one fatal outcome. Our findings demonstrate that ITPR3-related CID confers an increased susceptibility to EBV infection and EBV-associated complications, even life-threatening HLH. Based on these observations, we advocate for early consideration of hematopoietic stem cell transplantation (HSCT) as a potentially curative therapeutic strategy in this patient population.

## Materials and methods

### Genetic diagnosis

Peripheral blood and DNA samples from the patients were sent to MyGenostics (Beijing, China) for Whole-exome sequencing (WES) (14). Mutations in the *ITPR3* gene were confirmed by Sanger sequencing. The primers for the PCR: *ITPR3* Forward primers: CAGGAAGGGCCAAACCGCTAC, Reverse primers: GAGTCCTATCTTGCCCCAGTGAAT.

### NK cell function analysis

For the detection of CD107a expression on NK cells as our previously established protocol (15). Peripheral blood mononuclear cells (PBMCs) from patients and healthy controls were stimulated with K562 cells for 4 hours. Following incubation, the cells were retrieved and stained with CD3-Percp (Biolegend), CD56-PE (Biolegend), and CD107a-APC (Biolegend) at room temperature for 30 minutes. The stained cells were then analyzed using a BD celesta flow cytometer.

### Calcium flux analysis in T cells

Magnetic bead-sorted CD4+ T cells were subjected to calcium flux analysis following an established protocol (Blanco et al.) (13). Briefly, 1×10^6^ CD4+ T cells were pre-incubated with 0.6 μg/ml anti-CD3 monoclonal antibody (clone OKT3) for 10 minutes at 37°C in complete RPMI-1640 medium prior to acquisition. Baseline Fluo-4 fluorescence was recorded for 30 seconds under all experimental conditions. Cells were then stimulated with F(ab’)2 fragment (goat anti-mouse IgG, Jackson ImmunoResearch) for anti-CD3 preactivated cells, and then 1 μg/ml ionomycin (Sigma-Aldrich) as a positive control. Calcium flux kinetics were analyzed using FlowJo softwares.

### Lymphocyte proliferation and T follicular helper cell subset analysis

T cell proliferation were evaluated using a carboxyfluorescein succinimidyl ester (CFSE)-based dilution assay and circulating T follicular helper (Tfh) cell subsets analysis using flow cytometry according to established protocols (16). 1×10^6^ PBMCs were labeled with CFSE for cell dye and cell proliferation assessment. PHA or CD3/28 was employed to stimulate T lymphocytes, while F(ab)2’ IgM with CPG was used to stimulate B lymphocytes. The cells were incubated at 37°C, 5% CO2 for 72 hours.

### Plasmids

Full-length wild-type (WT) ITPR3 cDNA was obtained from Youbao Biotechnology, Changsha, China. Mutant ITPR3 cDNA was constructed by PCR mutagenesis and subcloned.

into the 7.1-pCMV-3×Flag vector. The sequence is sequenced for confirmation by Sangon Biotech (Shanghai, China). The overexpression experiment in 293T cells was performed according to our previously established protocol (17).

### Western blot experiment

293T cells or 1×10^6^ PBMCs were lysed with protein splitting mixture, and then supernatant was discarded by high-speed centrifugation to obtain protein. Protein were separated by concentrated gel and separation gel, with ITPR3 rabbit monoclonal antibody or Flag labeled, and infrared imager was used for detection and analysis.

### Diagnostic guidelines for hemophagocytic lymphohistiocytosis

This article adopts the diagnostic guidelines of HLH-2004 (18), and meets five of the eight criteria to consider diagnosing HLH. The specific content is shown in **Supplementary Table 1**.

## Results

### Case presentation

**Figure 1:**
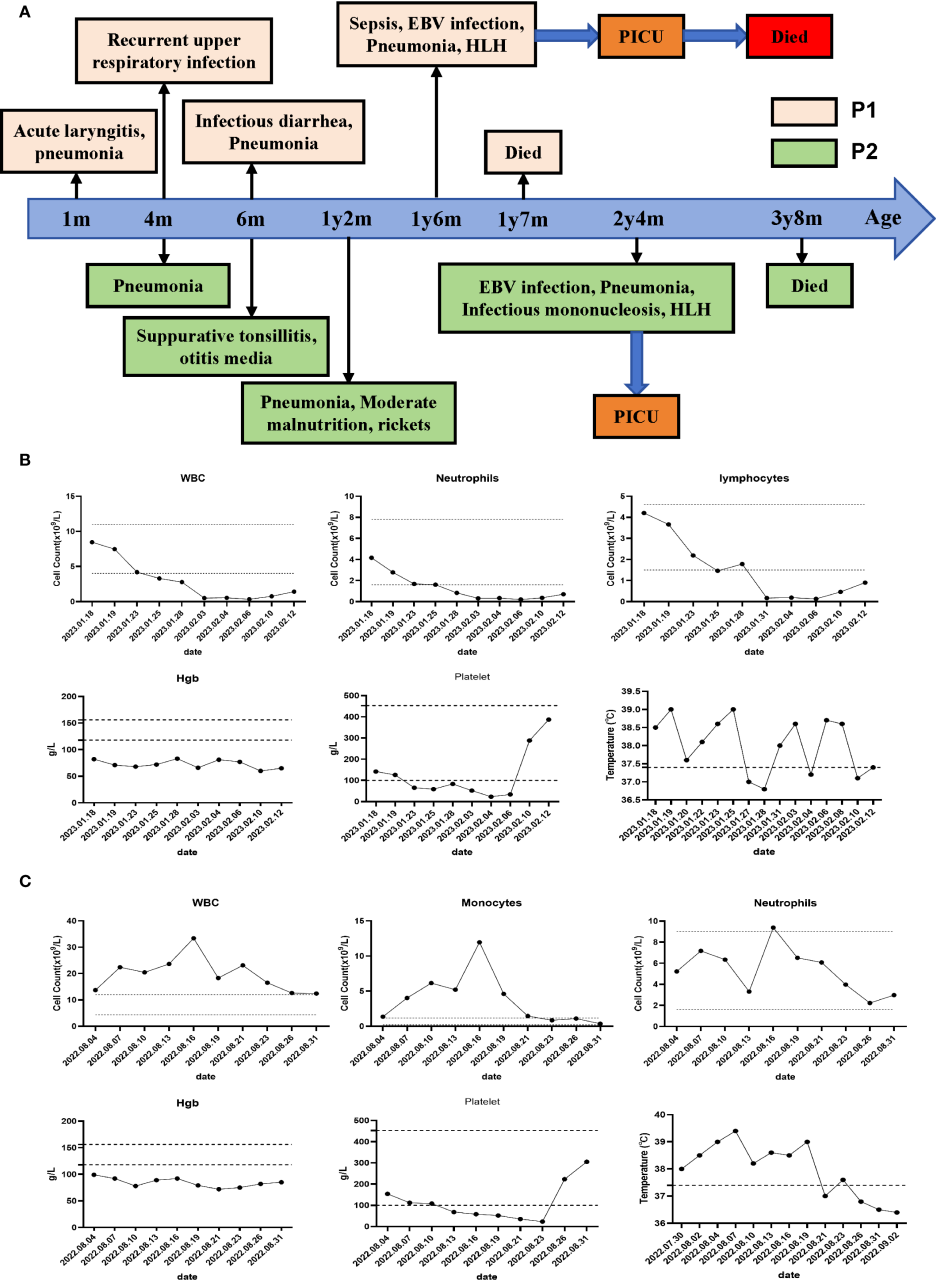
Clinical course and manifestations of the two patients. **(A)** Clinical course of the P1 and P2. **(B)** Peripheral hematological analysis of P1 and temperature monitoring during the disease course of P1 were performed. **(C)** Peripheral hematological analysis of P2 and temperature monitoring during the disease course of P2 were performed.

**Figure 2:**
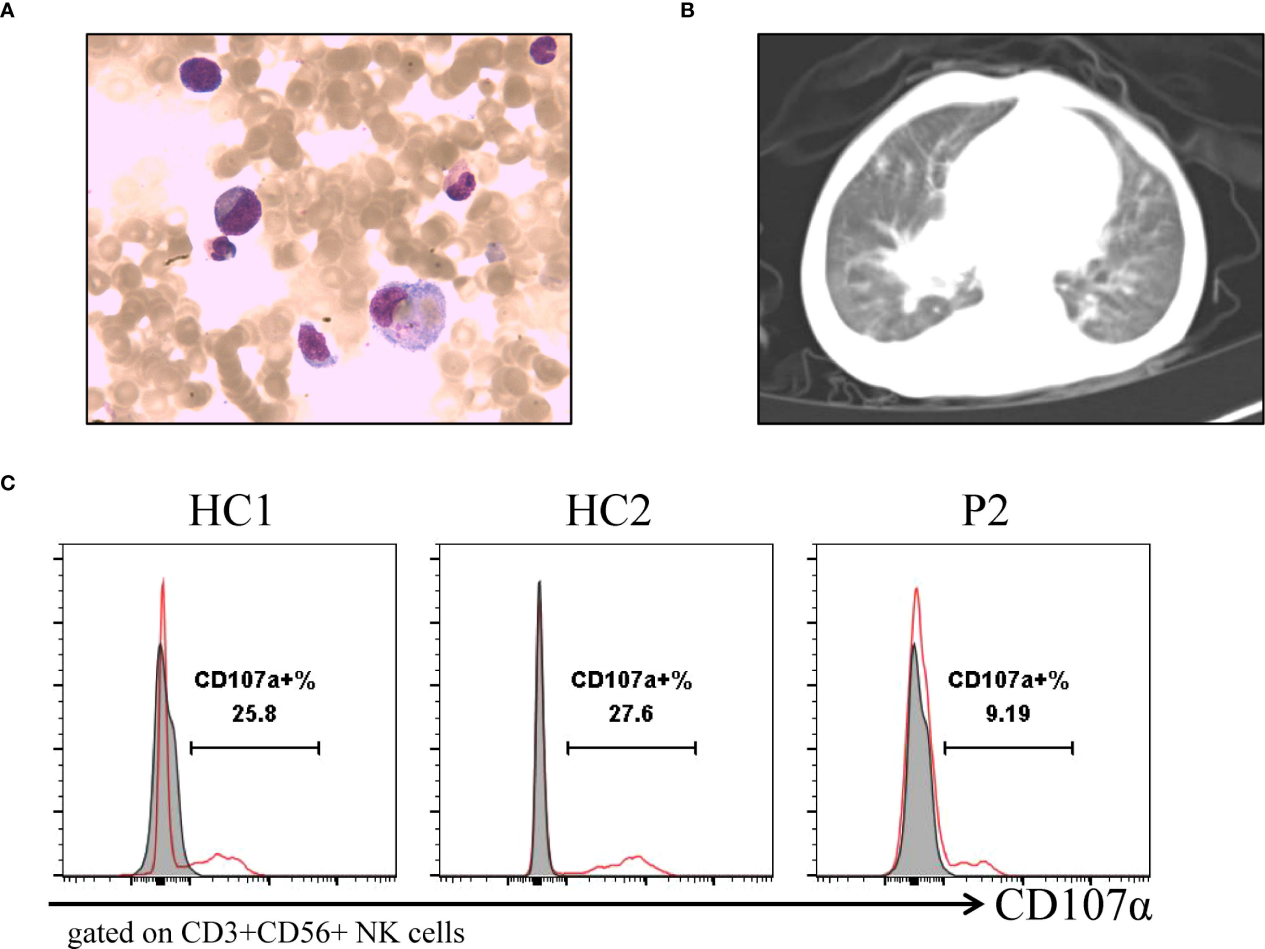
Clinical imaging and laboratory examinations of patients. **(A)** Bone marrow aspirates show hemophagocytic cells that engulf platelets, and red blood cells of P1. **(B)** Chest CT shows widespread lesions in both lungs of P2. **(C)** Flow cytometry detection of NK cell activity and CD107a analysis of degranulation function of P2.

Patient 1 (P1) was the first child of non-consanguineous Han Chinese parents, born at full term to healthy parents. His symptoms began in the neonatal period, requiring NICU admission due to acute laryngitis and severe pneumonia. Subsequently, he experienced frequent recurrent upper respiratory tract infections (2–4 episodes/month) and was hospitalized at 6 months of age for severe rotavirus gastroenteritis and streptococcal pneumonia (**Figure 1A**). The patient exhibited global developmental delay with significant retardation in both language and motor development. At 18 months, he was admitted with persistent fever, cough, hepatosplenomegaly, and lymphadenopathy. Admission evaluation showed he was conscious with pale complexion, no skin rash, but with facial and periorbital edema; vital signs included temperature 38.5 °C, respiratory rate 40/min, blood pressure 76/41 mmHg, and oxygen saturation 90%. Laboratory tests revealed: WBC 8.46×10^9^/L, HGB 82 g/L, PLT 142×10^9^/L; peripheral blood smear showed no abnormal erythrocyte or leukocyte morphology; ESR 64 mm/h; PCT 1.53 ng/mL; CRP 20.73 mg/L. Imaging studies demonstrated: chest CT revealed multiple pulmonary infectious lesions consistent with pneumonia; abdominal ultrasound showed mild hepatosplenomegaly, hypoechoic areas around the portal vein sagittal section, enlarged retroperitoneal lymph nodes, ascites, and bilateral cervical lymphadenopathy. Subsequently, he developed progressive cytopenia involving all three lineages (neutrophils, hemoglobin, and platelets), prompting further investigations for suspected HLH (**Figure 1B**). Coagulation and liver function tests revealed hypofibrinogenemia, elevated D-dimer, increased international normalized ratio, hypertriglyceridemia and elevated alanine aminotransferase (ALT). The cytokine profile revealed elevated levels of IL-6, IL-8, IL-10, and IFN-γ. Additionally, he exhibited hyperferritinemia and elevated soluble IL-2 receptor (sCD25) (**Table 1**). His natural killer (NK) cell count and activity were normal (**Supplementary Figure 1**). Bone marrow cytology demonstrated the presence of hemophagocytic macrophages (**Figure 2A**). Consequently, based on the HLH-2004 diagnostic criteria, the patient was diagnosed with HLH (meeting 7 out of 8 diagnostic criteria, including fever, hepatosplenomegaly, trilineage cytopenia, hypertriglyceridemia, hypofibrinogenemia, the presence of hemophagocytes in the bone marrow, hyperferritinemia, and elevated sCD25 levels) (**Supplementary Table 1**) (18). Subsequent investigations for infectious etiologies showed reactive EBV capsid antigen IgM (>160) with EBV nuclear antigen IgG at 5.31 U/mL, positive CMV capsid antigen IgM but negative CMV nuclear antigen IgG. Blood PCR confirmed high EBV viral load (3.13×10^6^ copies/mL, reference: <400 copies/mL). Blood cultures grew Staphylococcus aureus, while metagenomic next-generation sequencing (mNGS) of sputum detected bocavirus type 1 (432 sequence reads), Stenotrophomonas maltophilia (500 sequence reads), and human herpesvirus 4 EBV (2193 sequence reads) (19).

**Table 1:** Laboratory features of patient at diagnosis of HLH.

Laboratory features	Levels at diagnosis of P1	Levels at diagnosis of P2	References values
Fibrinogen	*0.56*	*0.26*	1.22-3.89g/L
Ferritin	**8234.27**	**1228.4**	27–375 ng/ml
Triglycerides	**2.21**	**1.44**	0.30–1.80mmol/L
sCD25	**8527**	**3524**	78–520 U/mL (> 2400)
D-Dimer	**3.40**	**2.18**	0–0.73 ng/mL
ALT	**99**	26	0–30.0 U/L
Albumin	36.8	22.8	39.0-54.0g/L
Total Bilirubin	12.5	**91.2**	0-26.0umol/L
Direct Bilirubin	**11.4**	**75.2**	0-10.0umol/L
INR	**1.23**	**1.4**	0.7-1.2s
LDH	168.5	205.6	145.0-300.0U/L
IgA	**6.89**	**7.95**	0.20-1.75 g/L
IgG	**24.8**	13.05	2.86-16.80 g/L
IgM	**3.60**	**2.57**	0.43-1.92 g/L
IgE	8.0	5	0-165 unti/L
C3	1.4	1.5	0.7-2.0g/L
C5	0.25	0.4	0.11-0.61g/L
IL-1β	6.56		0.00-12.4 pg/mL
IL-2	2.33	7.17	0.00-11.4 pg/mL
IL-4	1.24	4.22	0.00-12.9 pg/mL
IL-5	0.11		0.00-3.1 pg/mL
IL-6	**187,64**	**69.74**	0.00-20.0 pg/mL
IL-8	**68.63**		0.00-20.60 pg/mL
IL-10	**261.45**	**231.41**	0.00-5.90 pg/mL
IL-12p70	0.88		0.00-3.40 pg/mL
IL-17A	0.05	22.80	0.00-20.6 pg/mL
TNF-α	2.68	4.16	0.00-5.50 pg/mL
IFN-γ	**186.26**	**93.10**	0.00-17.30 pg/mL
IFN-α	**9.63**		0.00-8.50 pg/mL

IL, interleukin; TNF, tumor necrosis factor; IFN, interferon; bold, increased subpopulation.

P1 received combination therapy with glucocorticoids (methylprednisolone 100 mg/day for 3 days, then 50 mg/day for 6 days, followed by dexamethasone 4.5 mg/day for 3 days) and immunosuppressants (ruxolitinib 10 mg/day for 12 days, etoposide 35 mg/day for 2 days). Antimicrobial therapy included piperacillin-tazobactam plus meropenem, teicoplanin, sulfamethoxazole, and ganciclovir antiviral therapy, along with supportive care consisting of intravenous immunoglobulin (10 g/day for 3 days), transfusions of cryoprecipitate, fresh frozen plasma, red blood cells, and fresh platelets, as well as hepatocardiac protection with nutritional support. However, Patient P1 continued to exhibit persistent high fever, developed multi-organ involvement and coagulation disorders, and ultimately succumbed to the disease after treatment discontinuation by the parents against medical advice.

Patient 2 (P2), the second child born to non-consanguineous Han Chinese parents following an uncomplicated delivery. He has a healthy 10-year-old sister. Since early childhood, he has experienced recurrent infections including multiple episodes of pneumonia, sinusitis, otitis media, and tonsillitis (**Figure 1A**). He has also failed to meet numerous developmental and social milestones, demonstrating both language and motor delays, along with physical dysmorphic features including thin hair and nails, square skull configuration, moderate malnutrition, and pectus carinatum. At 2 years and 4 months of age, he presented with recurrent high fever and cough to an outside hospital where he was diagnosed with “bronchopneumonia and suppurative tonsillitis” and treated with azithromycin and penicillin. Due to persistent high fever accompanied by hepatosplenomegaly, cervical lymphadenopathy, and periorbital edema, he was transferred to our institution. Laboratory findings showed: WBC 37×10^9^/L, Hb 99 g/L, PLT 154×10^9^/L, lymphocytes 52.6%, monocytes 11.9×10^9^/L, CRP 18.4 mg/L, and ESR 3 mm/h (**Figure 1C**). Thoracoabdominal CT revealed bilateral pulmonary inflammation, pleural effusions, axillary and hilar lymphadenopathy, hepatosplenomegaly, mesenteric lymphadenopathy, and ascites (**Figure 2B**). Parotid and cervical ultrasound showed no significant parotid abnormalities but demonstrated multiple enlarged cervical lymph nodes. Peripheral blood smear showed increased atypical lymphocytes (28%). EBV serology was positive for both VCA-IgM and IgG, with quantitative PCR confirming high EBV viral load (5.58×10^4^ copies/mL, reference <400 copies/mL). Infectious mononucleosis was suspected.

Subsequently, the patient developed progressive bicytopenia (hemoglobin and platelets). Initially, serum ferritin levels were within normal range. However, the patient subsequently developed persistent high fever accompanied by hypofibrinogenemia despite normal triglyceride levels. Elevated cytokines including IL-6, IL-10, and IFN-γ indicated a hyperinflammatory state. Subsequent laboratory tests revealed elevated ferritin and sCD25, along with impaired NK-cell degranulation function (**Table 1**) (**Figure 2C**). The patient was ultimately diagnosed with infectious mononucleosis-associated HLH as he met at least 7 (including high fever, hepatosplenomegaly, reduced hemoglobin and platelets, hypofibrinogenemia, diminished NK-cell activity, hyperferritinemia, and elevated sCD25 levels) of the HLH-2004 diagnostic criteria one week after admission (**Supplementary Table 1**) (18). Additional etiological findings including coinfection with Coxsackievirus and Mycoplasma pneumoniae.

Patient P2 received glucocorticoid therapy (dexamethasone 5 mg/day for 6 days, subsequently reduced to 2.5 mg/day for an additional 6 days) combined with immunosuppressive treatment (etoposide 50 mg/dose administered twice at 4-day intervals). Adjunctive therapies included antiviral prophylaxis with ganciclovir tablets (50 mg twice daily orally for 12 days), supportive intravenous immunoglobulin (5 g/day from August 17-19), oral vitamin K_1_ supplementation, and transfusion therapy comprising cryoprecipitate, fresh frozen plasma, and packed red blood cells, along with hepatoprotective therapy (Atomeron) and nutritional support. Following this intensive treatment regimen, the patient demonstrated gradual defervescence and clinical improvement without developing neurological or other complications, achieving full recovery and discharge after two weeks of hospitalization. Regrettably, the patient succumbed to unknown causes at home one year later.

### Genetic diagnosis

**Figure 3:**
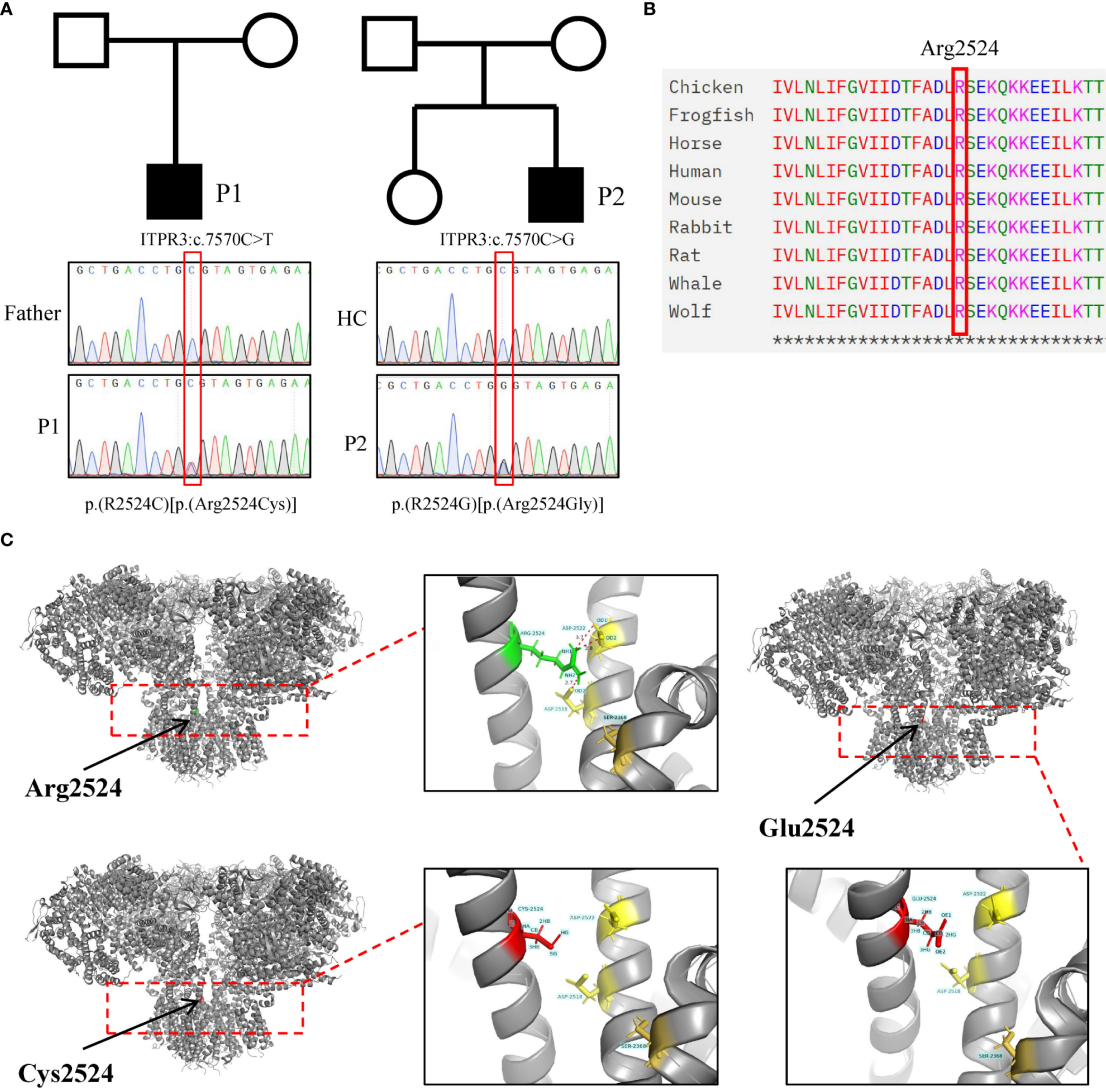
Identification and pathogenicity prediction of ITPR3 mutations in patients. **(A)** Sanger sequencing confirmed the ITPR3 mutation (c.7570C>T, p.Arg2524Cys) in P1 and ITPR3 mutation (c.7570C>G, p.Arg2524Gly) in P2. **(B)** Amino acid conservation analysis at the Arg2524 site. **(C)** Structural modeling and prediction of ITPR3 mutation sites were performed using AlphaFold2.

WES analysis of both patients revealed no pathogenic variants in known familial HLH-associated genes (such as PRF1, UNC13D, and STX11 et al.) (20). Notably, both patients harbored heterozygous *de novo* ITPR3 mutations that were absent in all unaffected family members. P1 carried the previously reported ITPR3: c.7570C>T, p.(Arg2524Cys) mutation, while P2 harbored a different nucleotide substitution at the identical position: c.7570C>G, p.(Arg2524Gly) (**Figure 3A**) (12, 13). Notably, both mutations occurred at the same site as previously documented cases, with the affected arginine residue being highly conserved across species (**Figure 3B**). Multiple prediction algorithms consistently classified these variants as pathogenic, supported by CADD scores of 34 and 35, respectively (**Table 2**). Using AlphaFold2, we further predicted that both R2524G and R2524C substitutions disrupt the interchain salt bridges normally formed by arginine, consistent with previous reports (**Figure 3C**). Based on these findings, we strongly suspect these mutations represent pathogenic variants responsible for our patients’ conditions.

**Table 2:** Prediction of ITPR3 mutation pathogenicity.

Position	*chr6:33660616*	*chr6:33660616*
Gene symbol	*ITPR3*	*ITPR3*
ref	C	C
alt	G	T
dbSNP	NA	NA
Consequence	Missense variant	Missense variant
HGVSc:nucleotide change	NM_002224.4:c.7570C>G	NM_002224.4:c.7570C>T
HGVSp: Protein change	NP_002215.2:p.Arg2524Gly	NP_002215.2:p.Arg2524Cys
SIFT(score)	Damaging(0)	Damaging(0)
PolyPhen (score)	Probably damaging(1)	Probably damaging(1)
CADD (phred score)	34	35
GERP++_RS	4.53	4.53
MutationTaster	Disease causing	Disease causing
PhastCons30way_mammalian	1	1

### Immunological analysis

**Figure 4:**
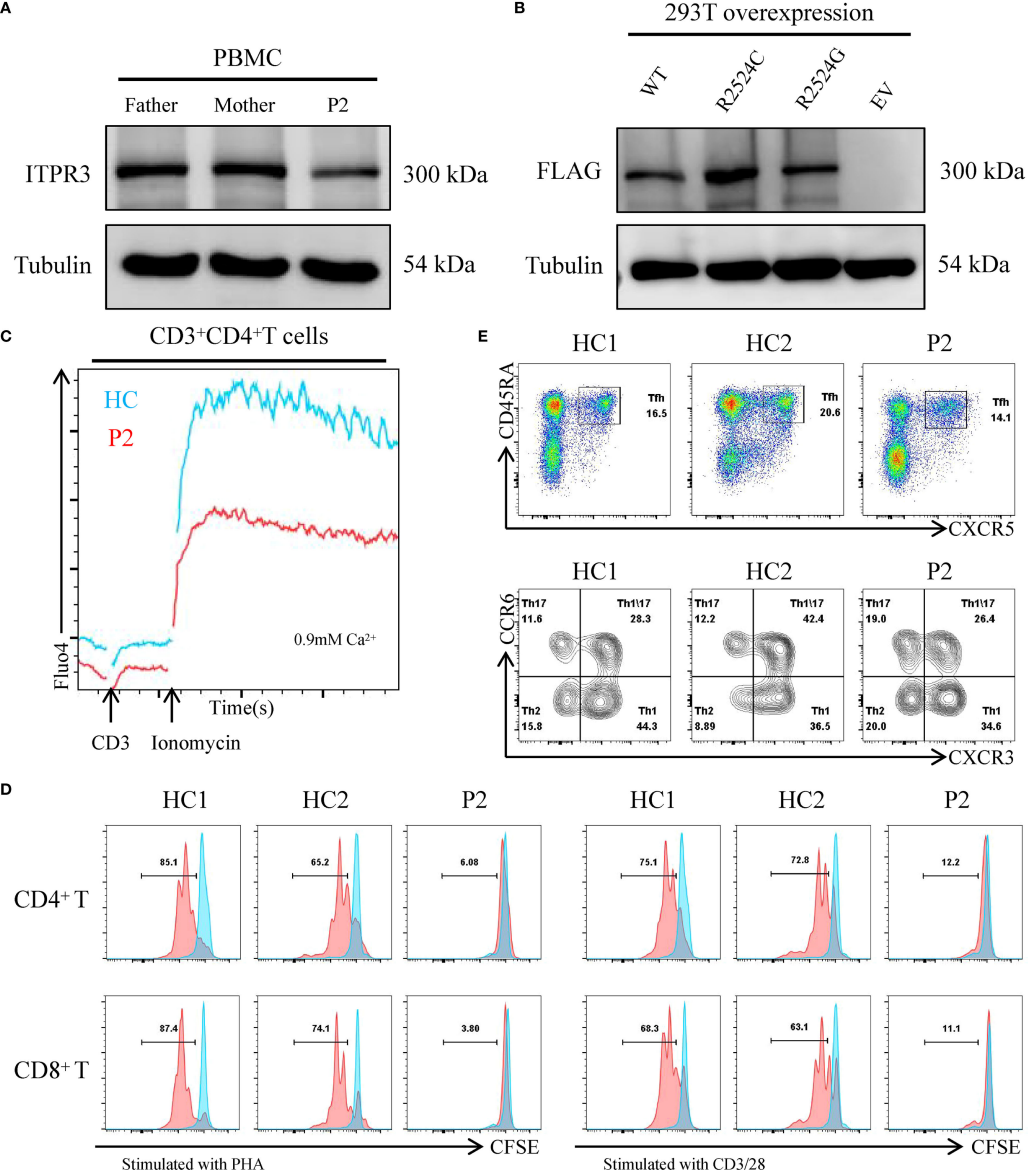
Expression and functional characterization of variant ITPR3 proteins. **(A)** ITPR3 protein expression analysis by western blot in primary PBMCs from P2, with tubulin serving as a loading control. **(B)** Comparative western blot analysis of WT and variant ITPR3 overexpression in transfected 293T cells. **(C)** Calcium flux analysis in primary T cells from P2, assessed by flow cytometry. **(D)** Proliferative capacity of CD4+ and CD8+ T cells from P2 following stimulation with either PHA or anti-CD3/CD28 antibodies. **(E)** Quantitative flow cytometric evaluation of CD4^+^CD45RA^+^CXCR5^+^ circulating T follicular helper (Tfh) cells and their subsets in P2.

Since we only obtained blood samples from P2, we subsequently focused our analysis on this patient. The ITPR3 protein expression level in P2’s PBMCs was comparable to that of his parents, and overexpression of the mutant ITPR3 protein in 293T cells showed no difference from WT, indicating that the mutation did not affect ITPR3 protein expression (**Figures 4A, B**). However, P2’s T cells exhibited significantly impaired Ca^2+^ influx following either TCR stimulation or ionomycin stimulation, confirming the pathogenicity of the c.7570C>G, p.(Arg2524Gly) mutation (**Figure 4C**). We then analyzed the immunological phenotypes of both patients. During HLH episodes, both patients showed normal or elevated immunoglobulin levels (**Table 1**). Absolute counts of all lymphocyte subsets were decreased, with reduced percentages of CD27^+^CD45RA^+^ cells (Naive) in both CD4^+^ and CD8^+^ T cell subsets, while CD27^+^CD45RA^-^ cells (CM) and CD27^-^CD45RA^-^ cells (EM) were increased (**Table 3**). These findings were consistent with the previously reported senescent T cell phenotype in ITPR3-mutated patients (12, 13). T cell proliferation assays in P2 demonstrated severely impaired proliferation upon either anti-CD3/CD28 or PHA stimulation (**Figure 4D**). However, the proportion of circulating Tfh cells and their subsets in P2’s peripheral blood showed no significant differences compared to healthy controls (**Figure 4E**). Collectively, these results confirm the pathogenicity of the ITPR3 variants in our patients.

**Table 3:** Immunological characteristics of the two patients.

Cell subpopulation	P1	Reference	P2	Reference
CD3+ cells %	68.8	53.88-72.87	73.3	66.9-83.1
CD3+CD8+ cells %	26.8	19.00-32.51	31.65	20.4-34.7
CD3+CD4+ cells %	41.5	24.08-42.52	40.85	33.19-47.85
CD19+ cells %	18.3	13.23-26.39	16.2	11.2-22.5
CD3-CD56+ cells %	12.3	7.21-20.90	11.4	6.25-19.36
CD4/CD8	1.55	0.90-2.13	1.29	0.97-2.31
CD3+ cells per uL	**619.2 (L)**	1400-8000	**1136.15 (L)**	1500-6500
CD3+CD8+ cells per uL	**241.2 (L)**	580-1735	**490.6 (L)**	520-1625
CD3+CD8+CD27+CD45RA+ cells % (Naive)	**24.7 (L)**	36.80-83.16	**25.3 (L)**	35.34-72.32
CD3+CD8+CD27+CD45RA- cells % (CM)	**37.1 (H)**	5.18-31.66	**33.8 (H)**	10.96-31.00
CD3+CD8+CD27-CD45RA- cells % (EM)	**19.3 (H)**	0.70-11.22	**29.9 (H)**	2.38-15.8
CD3+CD8+CD27-CD45RA+ cells % (TEMRA)	18.6	0.84-33.02	10.8	5.08-31.24
CD3+CD4+ cells per uL	**373.5 (L)**	902-2253	**633.2 (L)**	950-2500
CD3+CD4+CD27+CD45RA+ cells % (Naive)	**30.7 (L)**	46.14-84.40	**29.5 (L)**	39.5-66.26
CD3+CD4+CD27+CD45RA- cells % (CM)	43.2	13.88-48.12	45.7	25.34-49.90
CD3+CD4+CD27-CD45RA- cells% (EM)	**21.3 (H)**	0.94-6.46	**24.0 (H)**	4.68-15.70
CD3+CD4+CD27-CD45RA+ cells %(TEMRA)	**3.8 (H)**	0.0-1.36	0.8	0.00-1.54
CD19+ cells per uL	**164.7 (L)**	461-1456	**251.1 (L)**	300-1250
CD3–CD56+ cells per uL	**110.7 (L)**	270-1053	176.7	170-985

Values outside the reference range are shown in bold, with “L” indicating below the reference range and “H” indicating above the reference range.

## Discussion

Neurological disorders or inborn errors of immunity (IEI) associated with calcium signaling pathway defects are collectively termed CRAC channelopathies (3, 4). The identified calcium channelopathies encompass autosomal recessive loss-of-function (LOF) variants in STIM1, ORAI1, and CRACR2A, characterized by either early-onset severe combined immunodeficiency (SCID) or late-onset CID, accompanied by additional systemic manifestations including hypotonia, osteopetrosis, and anhidrotic ectodermal dysplasia (ED) with significant enamel defects (5–8). These patients typically demonstrate normal lymphocyte development but exhibit impaired proliferation, activation, and cytokine production, with most experiencing recurrent viral and bacterial infections that frequently necessitate HSCT for definitive treatment. ITPR3 encodes a subunit of IP3R, the ligand-gated Ca^2+^ channel on the endoplasmic reticulum membrane responsible for mediating SOCE (21). Recently, three independent groups reported ITPR3-associated CID accompanied by a multisystem syndrome resulting from autosomal recessive or dominant negative mutations (11–13). Notably, ITPR3 has previously been implicated in autosomal dominant Charcot-Marie-Tooth disease, although severe immunodeficiency was not described in these patients (10).

Here, we report two novel cases of ITPR3-associated disease carrying previously documented c.7570C>T, p.(Arg2524Cys) and a distinct nucleotide/amino acid substitution at the identical locus, c.7570C>G, p.(Arg2524Gly). Our findings substantiate the pathogenicity of both variants in our patients. First, both cases manifested severe recurrent bacterial/viral infections since infancy, accompanied by systemic features including developmental delay, alopecia, absent eyebrows, and pectus excavatum. Second, immunological analyses revealed decreased absolute lymphocyte counts and a senescent T-cell phenotype in both patients, consistent with ITPR3-related CID. Besides, both R2524G and R2524C substitutions disrupt the interchain salt bridges normally formed by arginine. Most significantly, although ITPR3 mutations did not affect protein expression levels, we observed severely impaired T-cell proliferation in P2 and defective Ca^2+^ influx following ionomycin stimulation, demonstrating the functional impact of ITPR3 mutations. These results are consistent with previous reports, suggesting that these two mutations may exhibit a dominant-negative effect (12, 13). Patients with CID are prone to severe viral infections, including EBV, with some potentially progressing to life-threatening HLH. Our patients developed EBV infection at a younger age with rapid progression to HLH, particularly P2 who developed HLH following infectious mononucleosis. Despite aggressive treatment, P1 ultimately succumbed to HLH complicated by multiple organ failure. Our data suggest that ITPR3 mutation-associated calcium channelopathy may be linked to HLH. The underlying mechanism could potentially involve either an indirect consequence of impaired immune control of EBV infection due to defective CRAC channel function leading to severe lymphocyte activation deficiency, or a combined outcome resulting from compromised exocytosis of cytotoxic lymphocyte granules. A similar association has also been observed in ORAI1-related calcium channelopathies (22).

The observed discrepancy in NK cell functional activity between the two patients is perplexing. It should be noted that Patient 1 underwent NK cell cytotoxicity testing at an external institution, which showed normal killing activity (as presented in **Supplementary Figure 1**). At that time, she had not yet met the diagnostic criteria for HLH. Regrettably, we were unable to retest this result. In contrast, Patient 2 was tested in our laboratory using NK cell degranulation analysis after meeting at least five HLH diagnostic criteria (including high fever, hepatosplenomegaly, reduced hemoglobin and platelets, hypofibrinogenemia, hyperferritinemia, and elevated sCD25 levels). The results demonstrated significantly reduced activity compared to healthy controls. Unfortunately, we could not perform further testing after HLH onset in either patient, as both had succumbed to the disease. Therefore, we speculate that the difference in results may be attributed to the distinct disease states at the time of testing, variations in the timing of assessments, or potentially intrinsic patient factors. This remains an important question worthy of further investigation. We recommend that future clinical management of such patients include monitoring of NK cell activity during HLH progression, particularly repeating the test after a certain interval for those with an initially negative screening result.

Another interesting observation is the partial overlap between CMT-associated ITPR3 mutations and CID-related mutations. For example, the ITPR3 hotspot mutation R2524C has been identified in both CID patients and those presenting with CMT only (10, 12, 13, 23). This suggests that the phenotypic differences are not attributable to distinct pathogenic mechanisms (e.g., gain-of-function or dominant-negative effects) nor to differences in the cellular populations affected by the mutation. We speculate that potential contributing factors may include reporting bias, modifier genes, epigenetic variations, as well as the patient’s ethnic background and living environment. Therefore, identifying additional cases and conducting careful longitudinal descriptions are essential to better understand the genetic and phenotypic heterogeneity of this disorder.

**Figure 5:**
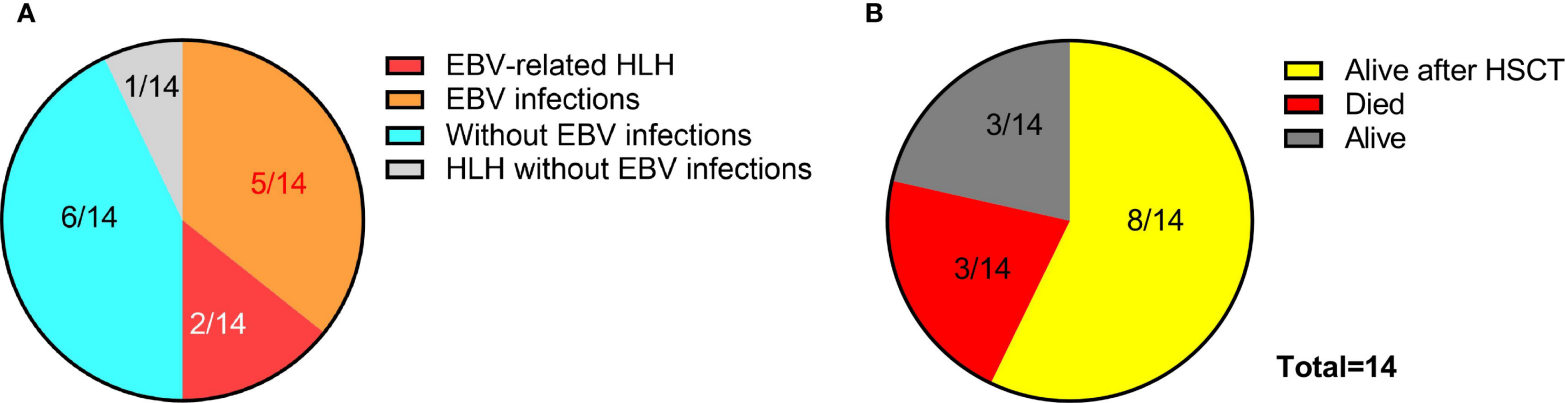
EBV infection profiles and clinical outcomes in patients with ITPR3 mutations. **(A)** Prevalence of EBV infection among 14 reported cases with ITPR3-related immunodeficiency. **(B)** Therapeutic interventions and survival outcomes in the 14-patient cohort.

We systematically reviewed 11 recently reported cases of ITPR3-mutated patients with immunodeficiency manifestations across three publications, and combined them with our two cases reported here plus an additional patient (designated P3, see **Supplementary Materials** for clinical history) carrying the pathogenic ITPR3 c.7570C>T, p.(Arg2524Cys) variant identified from our internal database, totaling 14 cases (11–13). Among these 14 patients, 7 developed EBV infection (7/14), presenting with lymphoma or lymphoproliferative disorders, while our two cases progressed to HLH. Notably, the fourth patient reported by Molitor et al. was diagnosed with an HLH-like syndrome at 1 year of age due to pancytopenia, hepatosplenomegaly, and generalized lymphadenopathy (as the patient didn’t meet full HLH diagnostic criteria at that time), though EBV infection status remained undetermined in this case (12). Among these 14 patients, 8 patients received HSCT (8/14) and achieved favorable outcomes, with 3 patients surviving to date without HSCT (3/14), while all 3 of our cases died (3/14) (**Figures 5A, B**) (11–13), highlighting the crucial importance of early and timely HSCT intervention.

## Conclusion

We present the fourth report of ITPR3 mutations causing combined immunodeficiency and further expand the clinical and molecular spectrum of this disorder. ITPR3 mutation-associated CID confers an increased susceptibility to EBV infection and carries a potential risk of progression to HLH. Early HSCT might be a curative intervention worth prioritizing in efforts to improve survival outcomes.

## Data Availability

The original contributions presented in the study are included in the article/**Supplementary Material**. Further inquiries can be directed to the corresponding authors.
