# Supplementary material for: Two cases of combined immunodeficiency with ITPR3 mutations presenting with life-threatening severe EBV-associated hemophagocytic lymphohistiocytosis
Source: Front Immunol. 2025 Sep 12;16:1653662. doi: 10.3389/fimmu.2025.1653662 (PMC12463974; doi:10.3389/fimmu.2025.1653662)
Supplement: Supplementary file 1 [file DataSheet1.pdf]

Supplementary Information of unprocessed original images for "Two Cases of Combined Immunodeficiency with ITPR3 Mutations Presenting with Life-Threatening Severe EBV-Associated Hemophagocytic Lymphohistiocytosis"

Figure 4A

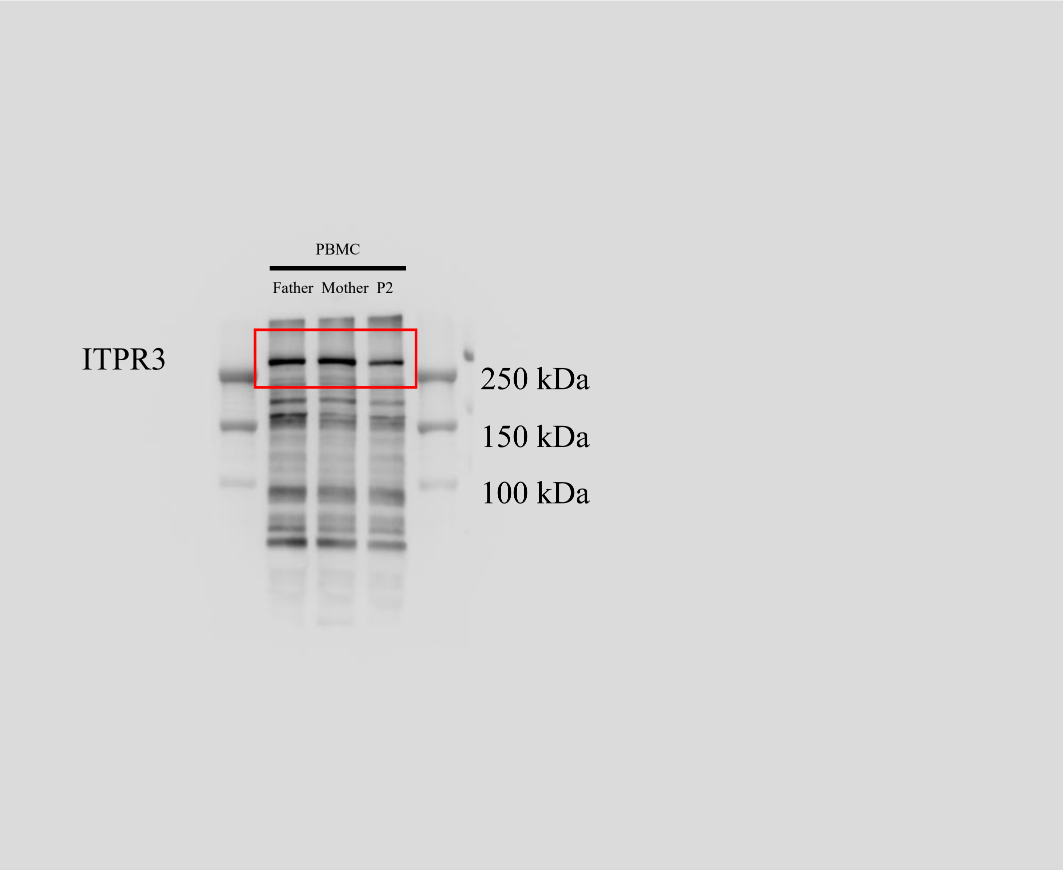

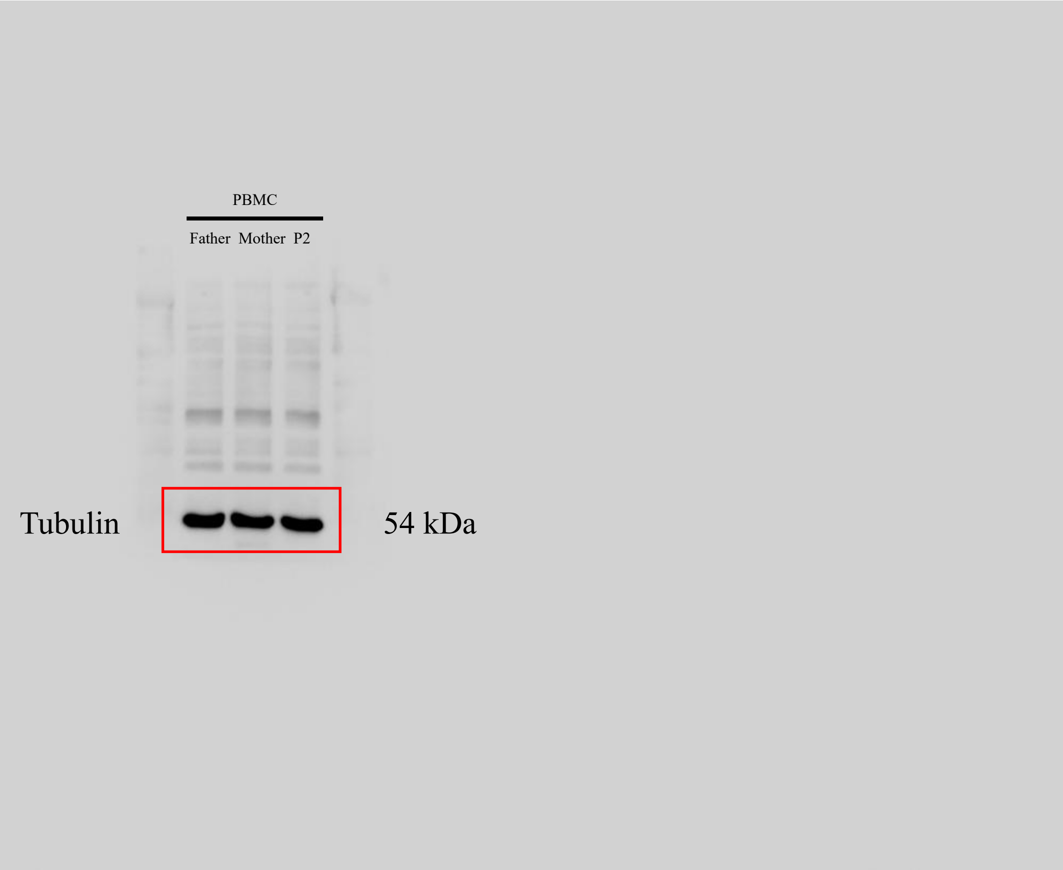

Figure 4B

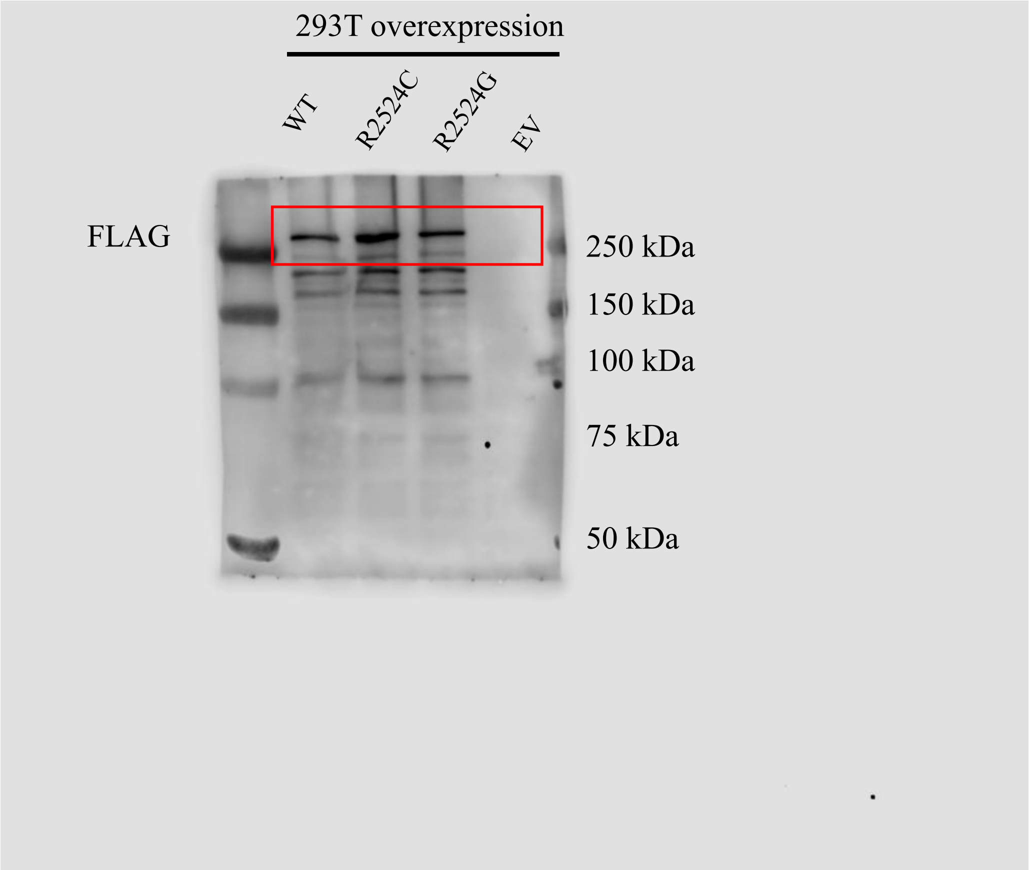
293T overexpression
WT
R2524C
R2524G
EV
FLAG
250 kDa
150 kDa
100 kDa
75 kDa
50 kDa

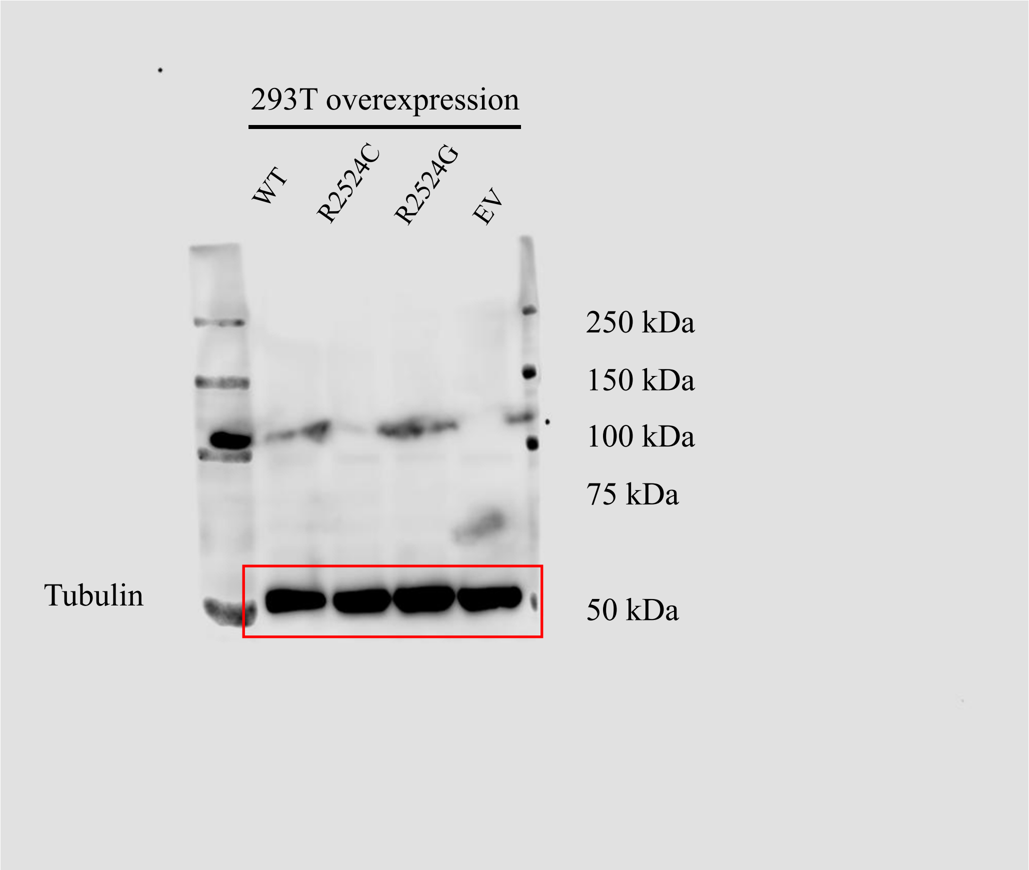
293T overexpression
WT
R2524C
R2524G
EV
250 kDa
150 kDa
100 kDa
75 kDa
Tubulin
50 kDa
