# Supplementary material for: Two cases of combined immunodeficiency with ITPR3 mutations presenting with life-threatening severe EBV-associated hemophagocytic lymphohistiocytosis
Source: Front Immunol. 2025 Sep 12;16:1653662. doi: 10.3389/fimmu.2025.1653662 (PMC12463974; doi:10.3389/fimmu.2025.1653662)
Supplement: Supplementary file 2 [file DataSheet2.docx]

### **Supplementary materials for “**Two Cases of Combined Immunodeficiency with ITPR3 Mutations Presenting with Life-Threatening Severe EBV-Associated Hemophagocytic Lymphohistiocytosis**”**

Lang Yu^1,2^, Yulin Li^1,2^, Liwen Zhang^1,2^, Wenhui Li^1,2,3^, Ming Sun^5^, Yunfei An^1,2,4^, Hao Xiong^5^, Peina Jin^6^, Xiaodong Zhao^1,2,4^

1. National Clinical Research Center for Child Health and Disorders, Ministry of Education Key Laboratory of Child Development and Disorders, Children’s Hospital of Chongqing Medical University, Chongqing, China
2. Chongqing Key Laboratory of Child Rare Diseases in Infection and Immunity, Children’s Hospital of Chongqing Medical University, Chongqing, China
3. Molecular Medicine Diagnostic and Testing Center, Chongqing Medical University, Chongqing, China
4. Department of Rheumatology & Immunology, Children’s Hospital of Chongqing Medical University, Chongqing, China
5. Department of Hematology, Wuhan Children's Hospital, Tongji Medical College, Huazhong University of Science and Technology, Wuhan, Hubei 430016, China.
6. PICU, The First Affiliated Hospital of Zhengzhou University, Zhengzhou, China.

Corresponding Authors:

Xiaodong Zhao, Department of Rheumatology and Immunology, Children’s Hospital of Chongqing

Medical University, No. 136, Zhongshan 2nd Road, Yuzhong District, Chongqing 400014, China;

email: zhaoxd530@aliyun.com;

Hao Xiong, Department of Hematology, Wuhan Children's Hospital, Tongji Medical College, Huazhong University of Science and Technology, Wuhan, Hubei 430016, China. email: [22587481@qq.com](mailto:22587481@qq.com).

Peina Jin, Department of PICU, The First Affiliated Hospital of Zhengzhou University, Zhengzhou, China. email: peinajin0103@163.com.

**Case presentation for another ITPR3 patient (P3)**

Patient P3, the first male child born to non-consanguineous Chinese parents, was delivered prematurely at 35^+2^ weeks of gestation. Immediately after birth, he was hospitalized for one month at a local hospital with neonatal respiratory distress syndrome, neonatal pneumonia, persistent pulmonary hypertension, atrial septal defect, and status post ductal ligation. At 3 months of age, he presented to our hospital with fever, cough, and dyspnea, and was diagnosed with: (1) severe pneumonia; (2) type I respiratory failure; (3) congenital heart disease (patent ductus arteriosus); (4) cholestatic hepatitis; (5) umbilical hernia; (6) right-sided pneumothorax; (7) bronchopulmonary dysplasia (BPD); (8) rubella virus infection; (9) cytomegalovirus infection; (10) herpes simplex virus infection; (11) thrombocytopenia; and (12) moderate anemia. At 5 months of age, he was readmitted with: (1) severe pneumonia; (2) type II respiratory failure; (3) cytomegalovirus infection; (4) BPD; (5) cholestatic hepatitis; (6) moderate anemia; (7) hepatomegaly (etiology unknown); (8) splenomegaly (etiology unknown); (9) umbilical hernia; (10) bilateral hydrocele; (11) congenital heart disease; and (12) suspected primary immunodeficiency. Pathological testing revealed positive IgM and IgG antibodies against rubella virus, cytomegalovirus, and herpes simplex virus types 1+2, and sputum culture was positive for Streptococcus pneumoniae. Despite aggressive antimicrobial therapy and supportive care, the patient ultimately succumbed to severe pneumonia and respiratory failure at another hospital. Immunological evaluations demonstrated: humoral immunity - IgG 7.99 g/L, IgA 1.46 g/L, IgM 0.27 g/L, complement C3 0.40 g/L, C4 0.10 g/L; complete blood count - WBC 6.1×10⁹/L, neutrophil count 4.87×10⁹/L, lymphocyte count 1.01×10⁹/L, RBC 3.10×10¹²/L, hemoglobin 95 g/L, platelets 191×10⁹/L. Phagocytic function testing (NBT test) was normal, though lymphocyte subsets and absolute counts were not evaluated. Whole-exome sequencing identified a de novo heterozygous ITPR3 mutation [c.7570C>T, p.(Arg2524Cys)] that was absent in both parents. This variant has been described in our study and previous literature, and the patient's clinical presentation definitively establishes its pathogenicity [1,2,3].

****References****

1. Neumann J, Van Nieuwenhove E, Terry LE, et al. Disrupted Ca2+ homeostasis and immunodeficiency in patients with functional IP3 receptor subtype 3 defects. *Cell Mol Immunol*. 2023;20(1):11-25. doi:10.1038/s41423-022-00928-4
2. Molitor A, Lederle A, Radosavljevic M, et al. A pleiotropic recurrent dominant ITPR3 variant causes a complex multisystemic disease. Sci Adv. 2024;10(37):eado5545. doi:10.1126/sciadv.ado5545
3. Blanco E, Camps C, Bahal S, et al. Dominant negative variants in ITPR3 impair T cell Ca2+ dynamics causing combined immunodeficiency. J Exp Med. 2025;222(1):e20220979. doi:10.1084/jem.20220979

**Supplementary table 1.** HLH-2004 diagnostic criteria in this patient.

**The diagnosis HLH can be established if one of either 1 or 2 below is fulfilled: P1 P2**

1. A molecular diagnosis consistent with HLH No No
2. Diagnostic criteria for HLH fulfilled (five out of the eight criteria below) Yes Yes

Fever 1/8 1/8

Splenomegaly 2/8 2/8

Cytopenias (affecting ≥ 2 of 3 lineages in the peripheral blood ): 3/8 3/8

Hemoglobin < 90 g/L (in infants < 4 weeks: hemoglobin < 100 g/L)

Platelets < 100 * 109/L

Neutrophils < 1.0 * 109/L

Hypertriglyceridemia and/or hypofibrinogenemia 4/8 4/8

Fasting triglycerides ≥ 3.0 mmol/L (i.e., ≥ 265 mg/dl)

Fibrinogen ≤ 1.5 g/L

Hemophagocytosis in bone marrow or spleen or lymph nodes. No evidence of malignancy 5/8

Low or absent NK-cell activity (according to local laboratory reference). 5/8

Ferritin ≥ 500 mg/L. 6/8 6/8

Soluble CD25 ≥ 2400 U/ml. 7/8 7/8

**Supplementary Figure 1**


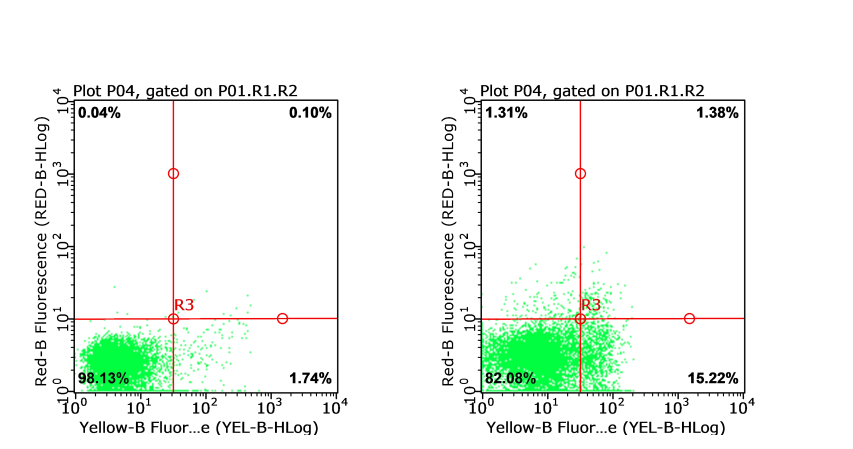


**Figure legend:** The left side shows the natural apoptosis results of the target cells alone (K562), while the right side displays the killing activity of the patient's NK cells against the target cells (percentage of Yellow B staining-positive cells, with a normal reference value of ≥15%).
